# Expanding Mental Health Horizons Across the Lifespan: Insights from the Congenital Heart Disease Population

**DOI:** 10.1192/j.eurpsy.2026.11067

**Published:** 2026-07-31

**Authors:** J. Brouillette, C. Houchi, M.-J. Marcil, P. Khairy, L. Rivard, M.-P. Dubé, M.-A. Chaix, F.-P. Mongeon, A. Dore, B. Mondésert, R. Ibrahim

**Affiliations:** 1 Psychiatry and Addiction, Université de Montréal; 2 Montreal Heart Institute; 3 Medecine, Université de Montréal, Montréal, Canada

## Abstract

**Introduction:**

Congenital heart defects affect approximately one in every 100 newborns. Nearly 60 years ago, most children with moderate or complex congenital heart disease (CHD) did not survive beyond their first year of life. However, recent medical and surgical advances have dramatically improved survival rates, resulting in a growing population of adults living with this chronic condition. Mental health disorders are among the most prevalent comorbidities in adults with CHD.

**Objectives:**

This study aimed to provide a biopsychosocial profile of this population, stratified by sex and age.

**Methods:**

We conducted a cross-sectional study including 264 adults with CHD, recruited at the Montreal Heart Institute (MHI) between 2019 and 2021. This study was approved by MHI Ethics and Research Committees and all patients provided written informed consent. Medical, lifestyle, and psychological data (including measures of anxiety, depression, personality, illness perception and coping) were collected using validated and emerging measures (*Hospital Anxiety and Depression Scale; Brief Illness Perception; Brief COPE; Personality Inventory for DSM-5—Brief Form*). Descriptive and comparative statistics were used to analyze differences across age and sex groups.

**Results:**

These findings highlight the importance of providing integrated physical and mental health care for the CHD population, particularly during the transition from pediatric to adult care. Sociodemographic factors and physical activity appear to be more plausible explanations for the variance in anxiety, while, surprisingly, the psychological variables studied did not vary by age group.

**Image 1:**
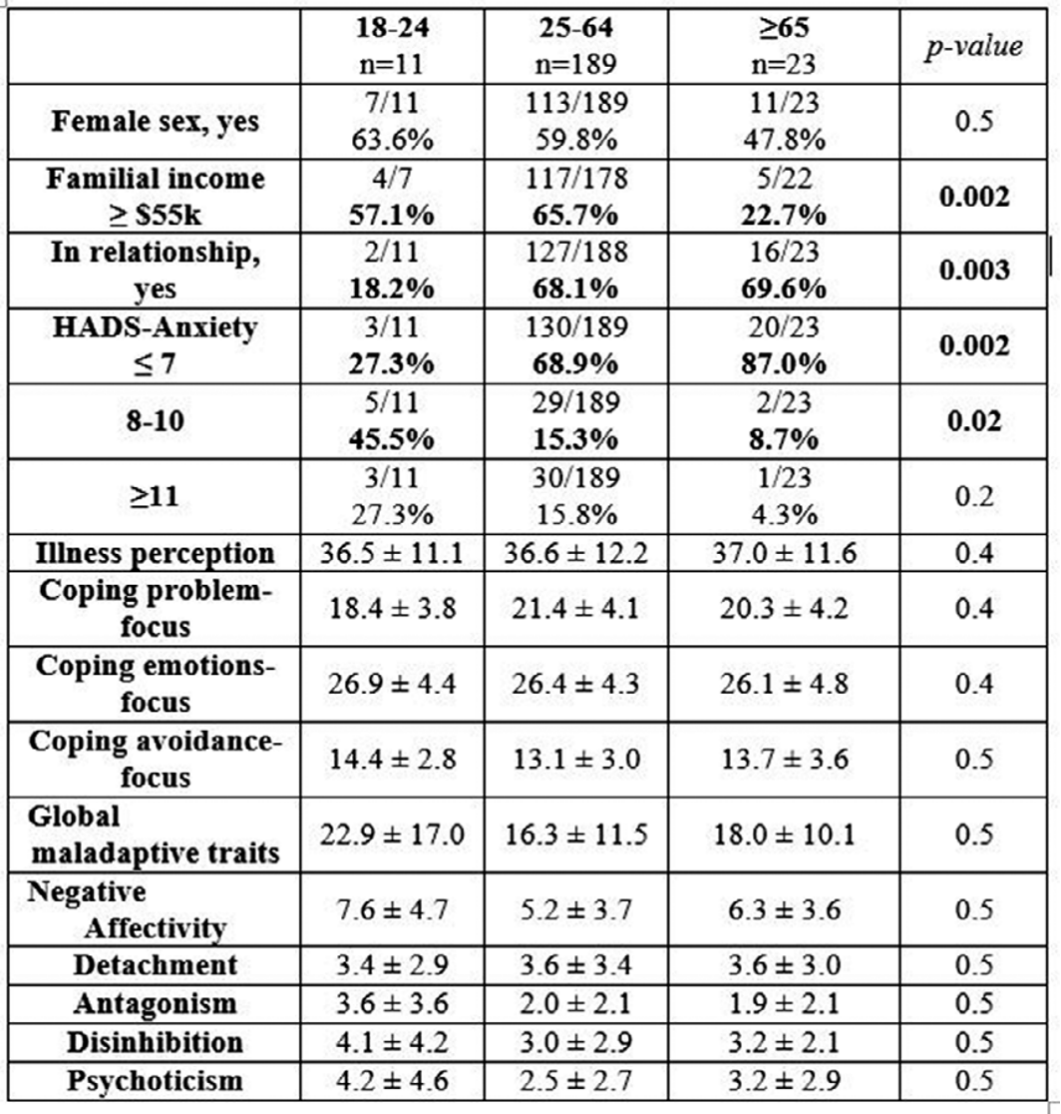
Image 1: Long description.

**Conclusions:**

These findings highlight the importance of providing integrated physical and mental health care for the CHD population, particularly during the transition from pediatric to adult care. Sociodemographic factors and physical activity appear to be more plausible explanations for the variance in anxiety, while, surprisingly, the psychological variables studied did not vary by age group.

**Disclosure of Interest:**

J. Brouillette Grant / Research support from: Dr. Brouillette reports financial support from the Montreal Heart Institute Foundation and Clinical Research Scholars - Junior 1 from the Fonds de Recherche du Québec - Santé (FRQS)., C. Houchi: None Declared, M.-J. Marcil: None Declared, P. Khairy: None Declared, L. Rivard: None Declared, M.-P. Dubé Consultant of: M-P. Dubé reports the following patents issued with Dalcor: Methods for the Treating or Preventing Cardiovascular Disorders and Lowering Risk of Cardiovascular Events (US20190070178A1), Genetic Markers for Predicting Responsiveness to Therapy with HDL-Raising or HDL Mimicking agent (US20170233812A1), and a patent pending with The Montreal Heart Institute and US Provisional: Methods for the using low dose colchicine after myocardial infraction (US20170233812A1). Also, she reports being an unpaid member of the board of directors of Educ’Alcool, a non-profit corporation, in addition to an equity interest in Dalcor Pharmaceuticals., M.-A. Chaix Grant / Research support from: Dr Chaix is supported by Junior 1 from the Fonds de Recherche du Québec - Santé (FRQS)., F.-P. Mongeon: None Declared, A. Dore: None Declared, B. Mondésert: None Declared, R. Ibrahim Consultant of: Dr. Ibrahim reports grants and consulting fees from Edward and Opsens, payments or honoraria from Abbott, Medtronic, Boston Scientific, Opsens, and Edwards and support for attending meetings and/or travel from Medtronic.

## Long descriptions

### Image 1: Long description

The table consists of 5 columns and 16 rows. The columns are: Variable, 18 to 24 (n equals 11), 25 to 64 (n equals 189), greater than or equal to 65 (n equals 23), and p-value.

Key findings include:

* Female sex, yes: 63.6 percent, 59.8 percent, 47.8 percent (p equals 0.5).

* Familial income greater than or equal to 55k: 57.1 percent, 65.7 percent, 22.7 percent (p equals 0.002).

* In relationship, yes: 18.2 percent, 68.1 percent, 69.6 percent (p equals 0.003).

* H A D S-Anxiety scores:

- Less than or equal to 7: 27.3 percent, 68.9 percent, 87.0 percent (p equals 0.002).

- 8 to 10: 45.5 percent, 15.3 percent, 8.7 percent (p equals 0.02).

- Greater than or equal to 11: 27.3 percent, 15.8 percent, 4.3 percent (p equals 0.2).

* Illness perception: 36.5 plus or minus 11.1, 36.6 plus or minus 12.2, 37.0 plus or minus 11.6 (p equals 0.4).

* Coping problem-focus: 18.4 plus or minus 3.8, 21.4 plus or minus 4.1, 20.3 plus or minus 4.2 (p equals 0.4).

* Coping emotions-focus: 26.9 plus or minus 4.4, 26.4 plus or minus 4.3, 26.1 plus or minus 4.8 (p equals 0.4).

* Coping avoidance-focus: 14.4 plus or minus 2.8, 13.1 plus or minus 3.0, 13.7 plus or minus 3.6 (p equals 0.5).

* Global maladaptive traits: 22.9 plus or minus 17.0, 16.3 plus or minus 11.5, 18.0 plus or minus 10.1 (p equals 0.5).

* Negative Affectivity: 7.6 plus or minus 4.7, 5.2 plus or minus 3.7, 6.3 plus or minus 3.6 (p equals 0.5).

* Detachment: 3.4 plus or minus 2.9, 3.6 plus or minus 3.4, 3.6 plus or minus 3.0 (p equals 0.5).

* Antagonism: 3.6 plus or minus 3.6, 2.0 plus or minus 2.1, 1.9 plus or minus 2.1 (p equals 0.5).

* Disinhibition: 4.1 plus or minus 4.2, 3.0 plus or minus 2.9, 3.2 plus or minus 2.1 (p equals 0.5).

* Psychoticism: 4.2 plus or minus 4.6, 2.5 plus or minus 2.7, 3.2 plus or minus 2.9 (p equals 0.5).

Navigate back to Image 1:.

